# The Sequenced Angiosperm Genomes and Genome Databases

**DOI:** 10.3389/fpls.2018.00418

**Published:** 2018-04-13

**Authors:** Fei Chen, Wei Dong, Jiawei Zhang, Xinyue Guo, Junhao Chen, Zhengjia Wang, Zhenguo Lin, Haibao Tang, Liangsheng Zhang

**Affiliations:** ^1^State Key Laboratory of Ecological Pest Control for Fujian and Taiwan Crops, College of Life Sciences, Fujian Provincial Key Laboratory of Haixia Applied Plant Systems Biology, Ministry of Education Key Laboratory of Genetics, Breeding and Multiple Utilization of Corps, Fujian Agriculture and Forestry University, Fuzhou, China; ^2^State Key Laboratory of Subtropical Silviculture, School of Forestry and Biotechnology, Zhejiang Agriculture and Forestry University, Hangzhou, China; ^3^Department of Biology, Saint Louis University, St. Louis, MO, United States

**Keywords:** angiosperm genomes, genome database, data sharing, big data, comparative genomics

## Abstract

Angiosperms, the flowering plants, provide the essential resources for human life, such as food, energy, oxygen, and materials. They also promoted the evolution of human, animals, and the planet earth. Despite the numerous advances in genome reports or sequencing technologies, no review covers all the released angiosperm genomes and the genome databases for data sharing. Based on the rapid advances and innovations in the database reconstruction in the last few years, here we provide a comprehensive review for three major types of angiosperm genome databases, including databases for a single species, for a specific angiosperm clade, and for multiple angiosperm species. The scope, tools, and data of each type of databases and their features are concisely discussed. The genome databases for a single species or a clade of species are especially popular for specific group of researchers, while a timely-updated comprehensive database is more powerful for address of major scientific mysteries at the genome scale. Considering the low coverage of flowering plants in any available database, we propose construction of a comprehensive database to facilitate large-scale comparative studies of angiosperm genomes and to promote the collaborative studies of important questions in plant biology.

## Introduction

**Figure 1:**
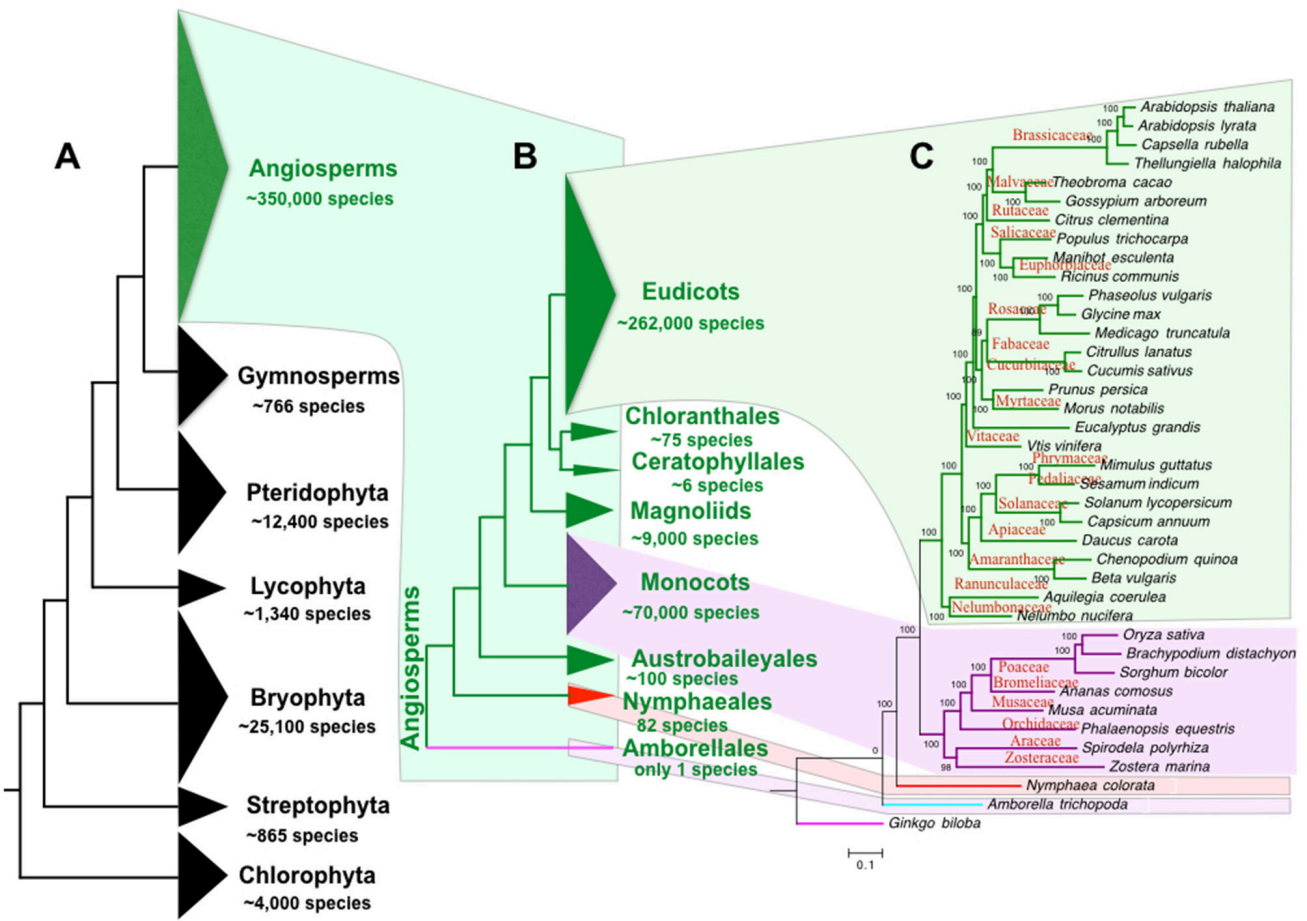
Phylogeny and species of green plants and angiosperms. **(A)** The tree of the plant life ranging from green algae to flowering plants. **(B)** The tree of angiosperm life. **(C)** Angiosperm species tree of representative references genomes. The angiosperm species number is estimated by www.theplantlist.org. Species number of other plant phylum is reported by Pryer et al. (2002). The number of Nymphaeales and other angiosperms species is summarized by Borsch et al. (2008) and Zeng et al. (2014), respectively.

The “green lineage” or the plant kingdom comprises ~4,000 chlorophyta algae, 865 charophyta algae, 25,100 bryophytes, 1,340 lycophytes, 12,400 pteridophytes, 766 gymnosperms (Pryer et al., 2002), and ~350,000 angiosperms (or flowering plants, estimated by www.theplantlist.org). Therefore, angiosperm is by far the most diverse group among all clades of the green lineage (Figure 1A). Originated from a single ancestor at about 167–199 mya (Bell et al., 2010), angiosperms have diverged into 8 extant clades, including Amborellales, Nymphaeales, Austrobaileyales, monocots, Magnoliids, Ceratophyllales, Chloranthales, and Eudicots (Zeng et al., 2014). Only one species is found in the basal branch angiosperm clade Amborellales, whereas the largest angiosperm clade eudicot contains ~262,000 species (Zeng et al., 2014; Figures 1B,C). Compared to other green lineage clades, the angiosperms play the most important roles in our human life. Our food, health, energy, materials, and environment largely depend on angiosperms. In addition, human culture is tightly linked to the utilization of angiosperms (Raskin et al., 2002). For example, early human cultures were shaped by the agriculture (Balick and Cox, 1996) including food production by rice and wheat, fruit gathering, wine fermentation, tea plantation, and flower culturing. Furthermore, angiosperms play important roles in the evolution of animal vision (Osorio and Vorobyev, 2008), taste (Li and Zhang, 2014), and olfactory sense (Niimura, 2012). The angiosperms also contributed greatly to the evolution of planet earth in the atmospheric cycle, water cycle, and the carbon cycle.

The *Science* editorial “So much more to know” raised 100 scientific questions to be answered and several of them are angiosperm-related (Hubble, 2005), such as: (1) How does a single somatic cell become a whole plant? (2) Why are some genomes really big and others quite compact? (3) What is all that “junk” doing in our genomes? (4) How did flowers evolve? Moreover, other important questions include (1) the origin of important innovations of the flowers and fruits, (2) the evolution of C4 and CAM photosynthesis, (3) the mechanisms of life style changes such as the epiphytes and parasites, (4) the genetic changes responsible for various ecological adaptations. Comparative genomics may hold the keys to these questions. The genomic sequences, bioinformatics tools, databases, and computing resources are essential infrastructures for comparative genomics.

Rich information can be identified in the angiosperm genomes, which contain various elements, including the genes, repetitive elements, centromeres. All angiosperms are paleo-polyploids (Van de Peer et al., 2017) and some harbor sex chromosomes (Charlesworth, 2016). A genome database is designed to store and present all the information. With the rapid development of bioinformatics, genome database has evolved from mere data storage platform to a novel discipline. Two textbooks focused on the genome databases have been published: “*Bioinformatics for Beginners: Genes, Genomes, Molecular Evolution, Databases and Analytical Tools*” (Choudhuri, 2014) and “*Genomes, Browsers and Databases: Data-Mining Tools for Integrated Genomic Databases*” (Schattner, 2008). Furthermore, new journals focused on database have been launched and database articles becoming more popular in various journals. A journal named as *Database: The Journal of Biological Databases and Curation* was launched in 2009 centered in the biological database. The annual special issues of database published by two high-impact journals, *Plant and Cell Physiology* and *Nucleic Acids Research*, have built a good reputation and become influential in the biological research community. In addition, database articles are also frequently published in other leading journals of plant science, such as *The Plant Cell* and *Molecular Plant*.

The main function of genome databases has evolved from data storage to online analysis, to lead the jigsaw puzzle in genome sequencing and resequencing projects. For example, Genome Database for Rosaceae (GDR) aims to host the genomes of all rosaceae species although only a few of their genomes have been sequenced. GDR integrated all the released rosaceae genomes and it is expected that more genome resequencing data would be added into the GDR. In the XIX International Botanical Congress (Shenzhen China, 2017), as a key part of the Earth BioGenome Project (EBP), a “10KP plan” was announced with an aim to sequence more than 10,000 genomes representing every major clade of plants and eukaryotic microbes.

In this review, we dedicate to provide readers the latest advances of angiosperm genome projects and database constructions. We discussed the pros and cons of three types of genome databases. We advocate a genome database for all the sequenced angiosperms for prompting data sharing. We also suggest a suit of standards for genome database establishment to boost the development of future databases. The future challenges in facing the biological big data were also discussed. We believe this review will shed new light on the development of angiosperm genome database in the near future.

## Genome and database overview

The first angiosperm genome database was launched in 2001 for the model plant *Arabidopsis thaliana* (Huala et al., 2001). Since 2001, various angiosperm genome databases have been developed synchronizing with the progress of sequencing projects of angiosperm genomes. The earliest angiosperm genome databases were designed as a repository of genome sequencing data. These databases have then evolved to serve as genome portals/hubs that integrate various genomic information, as well as web servers that provide online genomics analyses. These genome databases can be generally classified as three different types: single species database, comprehensive database, and clade-oriented database.

## The sequenced angiosperm genomes

As of August 31, 2017, the genomes of 236 angiosperm species have been completely sequenced. The list of sequenced angiosperm genomes and genome databases are provided in Table 1. The 236 species are found in 31 of the 64 angiosperm orders, thus nearly 50% of angiosperm orders have at least one genome sequenced. Most of them are plants of high economic importance or their wild relatives. More effort should be done on genome sequencing of more species that are important for study of evolutionary history of angiosperms such as magnoliids and basal angiosperms.

**Table 1:** A list of the public accessible plant genomes and their database construction status.

**Species**	**Order**	**Type**	**URL**
*Nymphaea colorata*	Nymphaeales	Ornamental	www.angiosperms.org
*Amborella trichopoda*	Amborellales	Wild	phytozome.jgi.doe.gov
*Elaeis guineensis*	Arecales	Economic	chibba.agtec.uga.edu/duplication/
*Phoenix dactylifera*	Arecales	Fruit	pgsb.helmholtz-muenchen.de
*Elaeis oleifera*	Arecales	Wild	
*Brachypodium distachyon*	Poales	Economic	plants.ensembl.org
*Eragrostis tef*	Poales	Economic	
*Eleusine coracana*	Poales	Food	
*Hordeum vulgare*	Poales	Food	phytozome.jgi.doe.gov
*Oryza indica*	Poales	Food	plants.ensembl.org
*Oryza sativa*	Poales	Food	rice.plantbiology.msu.edu/
*Setaria italica*	Poales	Food	phytozome.jgi.doe.gov
*Sorghum bicolor*	Poales	Food	gramene.org/
*Triticum aestivum*	Poales	Food	phytozome.jgi.doe.gov
*Triticum turgidum*	Poales	Food	gigadb.org
*Zea mays*	Poales	Food	plants.ensembl.org
*Secale cereale*	Poales	Food,	pgsb.helmholtz-muenchen.de
*Ananas comosus*	Poales	Fruit	phytozome.jgi.doe.gov
*Lolium perenne*	Poales	Ornamental	pgsb.helmholtz-muenchen.de
*Zizania latifolia*	Poales	Vegetable	
*Oryza punctats*	Poales	Weed	plants.ensembl.org
*Oryza rufipogon*	Poales	Weed	plants.ensembl.org
*Aegilops tauschii*	Poales	Wild	plants.ensembl.org
*Brachypodium stacei*	Poales	Wild	genome.jgi.doe.gov
*Dichanthelium oligosanthes*	Poales	Wild	genomevolution.org/CoGe
*Hordeum pubiflorum*	Poales	Wild	
*Leersia perrieri*	Poales	Wild	plants.ensembl.org
*Oropetium thomaeum*	Poales	Wild	genomevolution.orgauth.iplantc.org
*Oryza barthii*	Poales	Wild	plants.ensembl.org
*Oryza brachyantha*	Poales	Wild	plants.ensembl.org
*Oryza glaberrima*	Poales	Wild	plants.ensembl.org
*Oryza glumaepatula*	Poales	Wild	plants.ensembl.org
*Oryza glumipatula*	Poales	Wild	plants.ensembl.org
*Oryza meridionalis*	Poales	Wild	plants.ensembl.org
*Oryza nivara*	Poales	Wild	plants.ensembl.org
*Oryza longistaminata*	Poales	Wild	plants.ensembl.org
*Panicum hallii*	Poales	Wild	genomevolution.orgauth.iplantc.org
*Panicum virgatum*	Poales	Wild	genome.jgi.doe.gov
*Setaria viridis*	Poales	Wild	phytozome.jgi.doe.gov
*Triticum uratu*	Poales	Wild	gigadb.org
*Camptotheca acuminata*	Cornales	Ornamental, Economic	datadryad.org
*Musa balbisiana*	Zingiberales	Fruit	genomevolution.org
*Musa itinerans*	Zingiberales	Fruit	banana-genome-hub.southgreen.fr
*Ensete ventricosum*	Zingiberales	Ornamental	
*Musa acuminata*	Zingiberales	Ornamental	plants.ensembl.org
*Dendrobium catenatum*	Asparagales	Ornamental	
*Phalaenopsis equestris*	Asparagales	Ornamental	chibba.agtec.uga.edu/duplication/
*Xerophyta viscosa*	Pandanales	Wild	
*Lemna minor*	Alismatales	Economic	genomevolution.org
*Spirodela polyrhiza*	Alismatales	Economic	phytozome.jgi.doe.gov
*Zoysia japonica*	Alismatales	Ornamental	zoysia.kazusa.or.jp
*Zoysia matrella*	Alismatales	Ornamental	zoysia.kazusa.or.jp
*Zoysia pacifica*	Alismatales	Ornamental	zoysia.kazusa.or.jp
*Zostera marina*	Alismatales	Wild	phytozome.jgi.doe.gov
*Macleaya cordata*	Ranunculales	Economic	
*Aquilegia coerulea*	Ranunculales	Wild	genome.jgi.doe.gov
*Nelumbo nucifera*	Proteales	Ornamental	chibba.agtec.uga.edu/duplication
*Trifolium pratense*	Fabales	Drink	phytozome.jgi.doe.gov
*Lotus japonicus*	Fabales	Economic	chibba.agtec.uga.edu/duplication/
*Glycine soja*	Fabales	Food	soybase.org
*Lupinus angustifolius*	Fabales	Ornamental	
*Cajanus cajan*	Fabales	Vegetable	chibba.agtec.uga.edu/duplication/
*Cicer arietinum*	Fabales	Vegetable	chibba.agtec.uga.edu/duplication/
*Glycine max*	Fabales	Vegetable	plants.ensembl.org
*Medicago truncatula*	Fabales	Vegetable	www.medicagogenome.org/
*Phaseolus vulgaris*	Fabales	Vegetable	phytozome.jgi.doe.gov
*Vicia faba*	Fabales	Vegetable	
*Vigna angularis*	Fabales	Vegetable	viggs.dna.affrc.go.jp
*Vigna radiata*	Fabales	Vegetable	genomevolution.org/CoGe
*Vigna unguiculata*	Fabales	Vegetable	phytozome.jgi.doe.gov
*Arachis duranensis*	Fabales	Wild	www.peanutbase.org/
*Arachis ipaensis*	Fabales	Wild	www.peanutbase.org/
*Cicer reticulatum*	Fabales	Wild	www.coolseasonFoodlegume.org
*Trifolium subterraneum*	Fabales	Wild	
*Betula pendula*	Fabales	Wood, Economic	genomevolution.org
*Humulus lupulus*	Rosales	Drink	hopbase.cgrb.oregonstate.edu
*Cannabis sativa*	Rosales	Economic	genome.ccbr.utoronto.ca/cgi-bin/hgGateway
*Prunus avium*	Rosales	Food	ftp://ftp.bioinfo.wsu.edu
*Artocarpus camansi*	Rosales	Fruit	datadryad.org
*Ficus carica*	Rosales	Fruit	
*Fragaria nipponica*	Rosales	Fruit	
*Fragaria orientalis*	Rosales	Fruit	
*Fragaria vesca*	Rosales	Fruit	phytozome.jgi.doe.gov
*Fragaria* × *ananassa*	Rosales	Fruit	
*Malus domestica*	Rosales	Fruit	phytozome.jgi.doe.gov
*Morus notabilis*	Rosales	Fruit	morus.swu.edu.cn/morusdb
*Prunus persica*	Rosales	Fruit	phytozome.jgi.doe.gov
*Pyrus bretschneideri*	Rosales	Fruit	peargenome.njau.edu.cn
*Pyrus communis*	Rosales	Fruit	genomevolution.org
*Rubus occidentalis*	Rosales	Fruit	ftp://ftp.bioinfo.wsu.edu
*Ziziphus jujuba*	Rosales	Fruit	jujube.genomics.cn
*Prunus mume*	Rosales	Ornamental	chibba.agtec.uga.edu/duplication
*Rosa* × *damascena*	Rosales	Ornamental	
*Fragaria iinumae*	Rosales	Wild	
*Fragaria nubicola*	Rosales	Wild	
*Castanea mollissima*	Fagales	Fruit	hardwoodgenomics.org
*Juglans regia*	Fagales	Fruit	
*Betula nana*	Fagales	Wild	
*Quercus lobata*	Fagales	Wood	valleyoak.ucla.edu/genomesequence
*Cucumis melo*	Cucurbitales	Fruit	bioinformatics.psb.ugent.be/plaza/
*Cucumis sativus*	Cucurbitales	Vegetable	phytozome.jgi.doe.gov
*Lagenaria siceraria*	Cucurbitales	Vegetable	
*Momordica charantia*	Cucurbitales	Vegetable	
*Cephalotus follicularis*	Oxalidales	Economic	genomevolution.orgauth.iplantc.org
*Hevea brasiliensis*	Malpighiales	Economic	www4a.biotec.or.th/rubber
*Jatropha curcas*	Malpighiales	Economic	www.kazusa.or.jp/jatropha
*Populus tremula*	Malpighiales	Economic	plantgenie.org
*Populus tremulax*	Malpighiales	Economic	plantgenie.org
*Populus trichocarpa*	Malpighiales	Economic	phytozome.jgi.doe.gov
*Ricinus communis*	Malpighiales	Economic	phytozome.jgi.doe.gov
*Linum usitatissimum*	Malpighiales	Economic, Fiber	phytozome.jgi.doe.gov
*Manihot esculenta*	Malpighiales	Vegetable	phytozome.jgi.doe.gov
*Populus tremuloides*	Malpighiales	Wild	plantgenie.org
*Populus euphratica*	Malpighiales	Wild	
*Salix purpurea*	Malpighiales	Wild	phytozome.jgi.doe.gov
*Populus pruinosa*	Malpighiales	Wood	gigadb.org
*Populus deltoides*	Malpighiales	Wood	phytozome.jgi.doe.gov
*Eucalyptus camaldulensis*	Myrtales	Economic	www.kazusa.or.jp
*Punica granatum*	Myrtales	Food, Ornamental	
*Metrosideros polymorpha*	Myrtales	Wild	
*Eucalyptus grandis*	Myrtales	Wood	phytozome.jgi.doe.gov
*Theobroma cacao*	Malvales	Drink	www.cacaogenomedb.org/
*Gossypium raimondii*	Malvales	Economic	chibba.agtec.uga.edu/duplication/
*Corchirus olitorius*	Malvales	Fiber	
*Corchorus capsularis*	Malvales	Fiber	
*Gossypium arboreum*	Malvales	Fiber	www.cottongen.org
*Gossypium barbadense*	Malvales	Fiber	www.cottongen.org
*Gossypium hirsutum*	Malvales	Fiber	cgp.genomics.org.cn
*Hibiscus syriacus*	Malvales	Ornamental	hibiscus.kobic.re.kr/hibiscus.en
*Aquilaria agallochum*	Malvales	Wood	
*Brassica nigra*	Brassicales	Economic	brassicadb.org
*Camelina sativa*	Brassicales	Economic	
*Capsella orientalis*	Brassicales	Food	genomevolution.org
*Carica papaya*	Brassicales	Fruit	phytozome.jgi.doe.gov
*Lepidium meyenii*	Brassicales	Economic	ftp://202.203.187.112/genome/maca/
*Tarenaya hassleriana*	Brassicales	Ornamental	genomevolution.org/coge
*Barbarea vulgaris*	Brassicales	Vegetable	185.45.23.197:5080/Barbarea_data/
*Brassica juncea*	Brassicales	Vegetable	brassicadb.org
*Brassica napus*	Brassicales	Vegetable	gramene.org/
*Brassica oleracea*	Brassicales	Vegetable	plants.ensembl.org
*Brassica rapa*	Brassicales	Vegetable	brassicadb.org/brad/
*Capsella bursa-pastoris*	Brassicales	Vegetable	
*Capsella rubella*	Brassicales	Vegetable	phytozome.jgi.doe.gov
*Moringa oleifera*	Brassicales	Vegetable	ftp://202.203.187.112/genome/lamu/
*Raphanus sativus*	Brassicales	Vegetable	radish.kazusa.or.jp/index.html
*Thlaspi arvense*	Brassicales	Vegetable	
*Aethionema arabicum*	Brassicales	Wild	brassicadb.org
*Arabidopsis halleri*	Brassicales	Wild	phytozome.jgi.doe.gov
*Arabidopsis lyrata*	Brassicales	Wild	bioinformatics.psb.ugent.be/plaza/
*Arabidopsis thaliana*	Brassicales	Wild	www.arabidopsis.org/index.jsp
*Arabis alpina*	Brassicales	Wild	arabis-alpina.org
*Arabis montbretiana*	Brassicales	Wild	
*Arabis nordmanniana*	Brassicales	Wild	
*Boechera stricta*	Brassicales	Wild	genome.jgi.doe.gov
*Capsella grandiflora*	Brassicales	Wild	genomevolution.org
*Cardamine hirsuta*	Brassicales	Wild	chi.mpipz.mpg.de
*Eutrema salsugineum*	Brassicales	Wild	phytozome.jgi.doe.gov
*Leavenworthia alabamica*	Brassicales	Wild	brassicadb.org
*Raphanus raphanistrum*	Brassicales	Wild	genomevolution.org/coge
*Sisymbrium irio*	Brassicales	Wild	brassicadb.org
*Thellungiella parvula*	Brassicales	Wild	thellungiella.org/data/
*Citrus grandis*	Sapindales	Food,	citrus.hzau.edu.cn
*Citrus ichangensis*	Sapindales	Food,	citrus.hzau.edu.cn
*Citrus medica*	Sapindales	Food	citrus.hzau.edu.cn
*Citrullus lanatus*	Sapindales	Fruit	chibba.agtec.uga.edu/duplication
*Citrus clementina*	Sapindales	Fruit	phytozome.jgi.doe.gov
*Citrus sinensis*	Sapindales	Fruit	phytozome.jgi.doe.gov
*Dimocarpus longan*	Sapindales	Fruit	gigadb.org
*Atalantia buxifolia*	Sapindales	Wild	citrus.hzau.edu.cn
*Azadirachta indica*	Sapindales	Wild	
*Vitis vinifera*	Vitales	Fruit	plants.ensembl.org
*Vitis aestivalis*	Vitales	Fruit	
*Vitis cinerea* × *Vitis riparia*	Vitales	Fruit	
*Rhodiola crenulata*	Saxifragales	Economic	gigadb.org
*Kalanchoe fedtschenkoi*	Saxifragales	Ornamental	phytozome.jgi.doe.gov
*Kalanchoe laxiflora*	Saxifragales	Ornamental	phytozome.jgi.doe.gov
*Kalanchoe marnieriana*	Saxifragales	Ornamental	genomevolution.org
*Chenopodium pallidicaule*	Caryophyllales	Food	
*Chenopodium quinoa*	Caryophyllales	Food	phytozome.jgi.doe.gov
*Fagopyrum esculentum*	Caryophyllales	Food	buckwheat.kazusa.or.jp
*Amaranthus hypochondriacus*	Caryophyllales	Ornamental	phytozome.jgi.doe.gov
*Dianthus caryophyllus*	Caryophyllales	Ornamental	carnation.kazusa.or.jp
*Drosera capensis*	Caryophyllales	Ornamental	
*Beta vulgaris*	Caryophyllales	Vegetable	plants.ensembl.org
*Spinacia oleracea*	Caryophyllales	Vegetable	genomevolution.org
*Amaranthus tuberculatus*	Caryophyllales	Weed	
*Chenopodium suecicum*	Caryophyllales	Wild	
*Silene latifolia subsp. alba*	Caryophyllales	Wild	
*Eichhornia paniculata*	Commelinales	Wild	
*Camellia sinensis*	Ericales	Drink	www.plantkingdomgdb.com
*Actinidia chinensis*	Ericales	Fruit	bdg.hfut.edu.cn
*Diospyros lotus*	Ericales	Fruit	chibba.agtec.uga.edu/duplication/
*Vaccinum macrocarpon*	Ericales	Fruit	
*Primula veris*	Ericales	Wild	
*Helianthus annuus*	Asterales	Economic	www.sunflowergenome.org
*Silybum marianum*	Asterales	Economic	
*Carthamus tinctorius*	Asterales	Oil	
*Lactuca sativa*	Asterales	Vegetable	phytozome.jgi.doe.gov
*Conyza canadensis*	Asterales	Weed	
*Panax notoginseng*	Apiales	Economic	ftp://202.203.187.112/genome/sanqi
*Daucus carota*	Apiales	Vegetable	phytozome.jgi.doe.gov
*Nicotiana benthamiana*	Solanales	Economic	genomevolution.org
*Nicotiana tabacum*	Solanales	Economic	solgenomics.net
*Ipomoea nil*	Solanales	Ornamental	
*Nicotiana sylvestris*	Solanales	Ornamental	solgenomics.net
*Petunia inflata*	Solanales	Ornamental	genomevolution.org
*Petunia integrifolia*	Solanales	Ornamental	genomevolution.org
*Capsicum annuum*	Solanales	Vegetable	chibba.agtec.uga.edu/duplication
*Solanum lycopersicum*	Solanales	Vegetable	phytozome.jgi.doe.gov
*Solanum melongena*	Solanales	Vegetable	genomevolution.org
*Solanum pimpinellifolium*	Solanales	Vegetable	solgenomics.net
*Solanum tuberosum*	Solanales	Vegetable	phytozome.jgi.doe.gov
*Ipomoea trifida*	Solanales	Wild	sweetpotato-garden.kazusa.or.jp
*Nicotiana attenuata*	Solanales	Wild	
*Nicotiana otophora*	Solanales	Wild	
*Nicotiana tomentosiformis*	Solanales	Wild	solgenomics.net
*Petunia axilaris*	Solanales	Wild	genomevolution.org
*Solanum arcanum*	Solanales	Wild	
*Solanum commersonii*	Solanales	Wild	solgenomics.net
*Solanum habrochaites*	Solanales	Wild	
*Solanum pennellii*	Solanales	Wild	solgenomics.net
*Sesamum indicum*	Lamiales	Economic	www.ocri-genomics.org/Sinbase/index.html
*Dorcoceras hygrometricum*	Lamiales	Economic	
*Mentha longifolia*	Lamiales	Economic	langelabtools.wsu.edu/mgr/organism/Mentha/longifolia
*Ocimum tenuiflorum*	Lamiales	Economic	
*Pogostemon cablin*	Lamiales	Economic	ftp://202.203.187.112
*Salvia miltiorrhiza*	Lamiales	Economic	ftp://202.203.187.112
*Erythranthe guttata*	Lamiales	Ornamental	
*Fraxinus excelsior*	Lamiales	Ornamental	www.ashgenome.org
*Mimulus guttatus*	Lamiales	Ornamental	phytozome.jgi.doe.gov
*Antirrhinum majus*	Lamiales	Ornamental, Economic	genomevolution.org
*Genlisea aurea*	Lamiales	Wild	
*Utricularia gibba*	Lamiales	Wild	chibba.agtec.uga.edu/duplication/
*Coffea canephora*	Gentianales	Drink	genomevolution.org
*Catharanthus roseus*	Gentianales	Economic	plantgenomics.msu.edu
*Rhazya stricta*	Gentianales	Economic	

*The blank link of 58 species suggest these genome information were stored in NCBI-Genome column*.

After the completion of the genome sequencing, an urgent issue is to share the genome data with the research community immediately after the genome release. The importance of data sharing is well recognized because it expands the impact of these valuable sequence data and promotes collaboration. A good genome database should meet two criteria: (i) integration of various types of genomic data, and (ii) providing genome analysis tools.

## Genome database for a single species

Among the 236 sequenced angiosperm genomes, only a few of them have a well-constructed customized database (Table 1) to host its various genome information. 58 genomes are only stored at NCBI Genome without a customized database. The genome databases of model plants *Arabidopsis* and rice (*Oryza sativa*) appear to be most well constructed. The most popular *Arabidopsis* database is the Arabidopsis Information Resource (TAIR, www.arabidopsis.org) (Garcia-Hernandez et al., 2002). TAIR provides updated genome sequence (currently V10) and various genomic information, including SNP, transposons, genes, gene families, gene annotations, gene names, proteins, and mutant orderings. Multiple web-integrated bioinformatics tools are also provided by TAIR. For examples, BLAST, WU-BLAST, FASTA, Gbrowse, Synteny Viewer, Seqviewer, Motif analysis, and Chromosome Map tool are powerful for visualization and comparative studies of genes and genome sequence at different scales. Pathway maps provide predicted gene interaction information, and has gained its popularity for the rapid development of metabolic and metabolomics researches. Other tools include Mapviewer, Metabolic Pathways, N-browse, Patmatch, VxInsight, Java Tree View, Bulk Data Retrieval, Gene Symbol Registry, and Textpresso Full Text. ARAPOT (www.araport.org) is also an important *Arabidopsis* genome database that provides updated genome sequence (currently V11), various gene information and protein interaction networks. However, another *Arabidopsis* database (Schoof et al., 2002) is no longer accessible. Among the web-integrated bioinformatics tools, such as those in TAIR as an example, BLAST, WU-BLAST, FASTA, Gbrowse, Synteny Viewer, Seqviewer, Motif analysis, and Chromosome Map tool were developed for visualize and compare genes and genome sequence at various scales. Patmatch and Bulk Data Retrieval tools help users to fetch data from servers. Pathway maps provide predicted gene interaction information, and are becoming popular nowadays for the rapid development of metabolic and metabolomics researches. Besides, the other tools are good complementary to various purposes.

However, unlike TAIR, most species-specific genome databases do not offer a rich collection of bioinformatic tools. For example, the pear (*Pyrus bretschneideri*.) genome database (peargenome.njau.edu.cn) only provide data download. The ash tree (*Hymenoscyphus fraxinea* and *Agrilus planipennis*) genome database (ashgenome.org/) includes the BLAST, Jbrowse tools, and data download service. The jujube (*Ziziphus jujuba*) genome database (jujube.genomics.cn/page/species/index.jsp) has tools such as gene search, Mapview and BLAST. The limited availability of bioinformatic tools may have reduced the popularity and usability of these databases.

Constrution a comprehensive database requires the knowledge of databases and a plethora of database and web programming languages such as Java, HTML, PHP, MySQL, Python, Perl et al., which are not the expertise of most experimental biologists. Most of none-model plant genomes are sequenced by experimental biologists, which is probably the main factor for the different levels of functionalities among species-specific genome databases. Three problems are constantly encountered for species-specific genome databases. First, these databases are often constructed by outsourcing companies, or by one of the bioinformatics graduate student/staff. The cost of such database is usually low and less time consuming. However, the content of these databases is usually rarely or never updated, probably due to the expiration of the service contract with outsourcing companies or departures of graduate students/post-doctoral scholars. For this reason, many species-specific genome database are unstable and eventually become inactivated. For instance, the databases of Mei and pineapple are no longer accessible (only accessible for several months after the release of genomes). Although accessing some databases is not convenient, a few species have more than one genome database. Second, the visual design of these databases usually does not match to those comprehensive databases. The findability and accessibility are usually limited because the users of a species-specific genome database are usually limited to those who work on the same species (Adam-Blondon et al., 2016). Third, with the rapid development of sequencing technology, many more genomes have been sequenced but no customized genome database was built for these genomes, although they may be economically or evolutionarily important.

## Comprehensive databases for various angiosperm species

**Figure 2:**
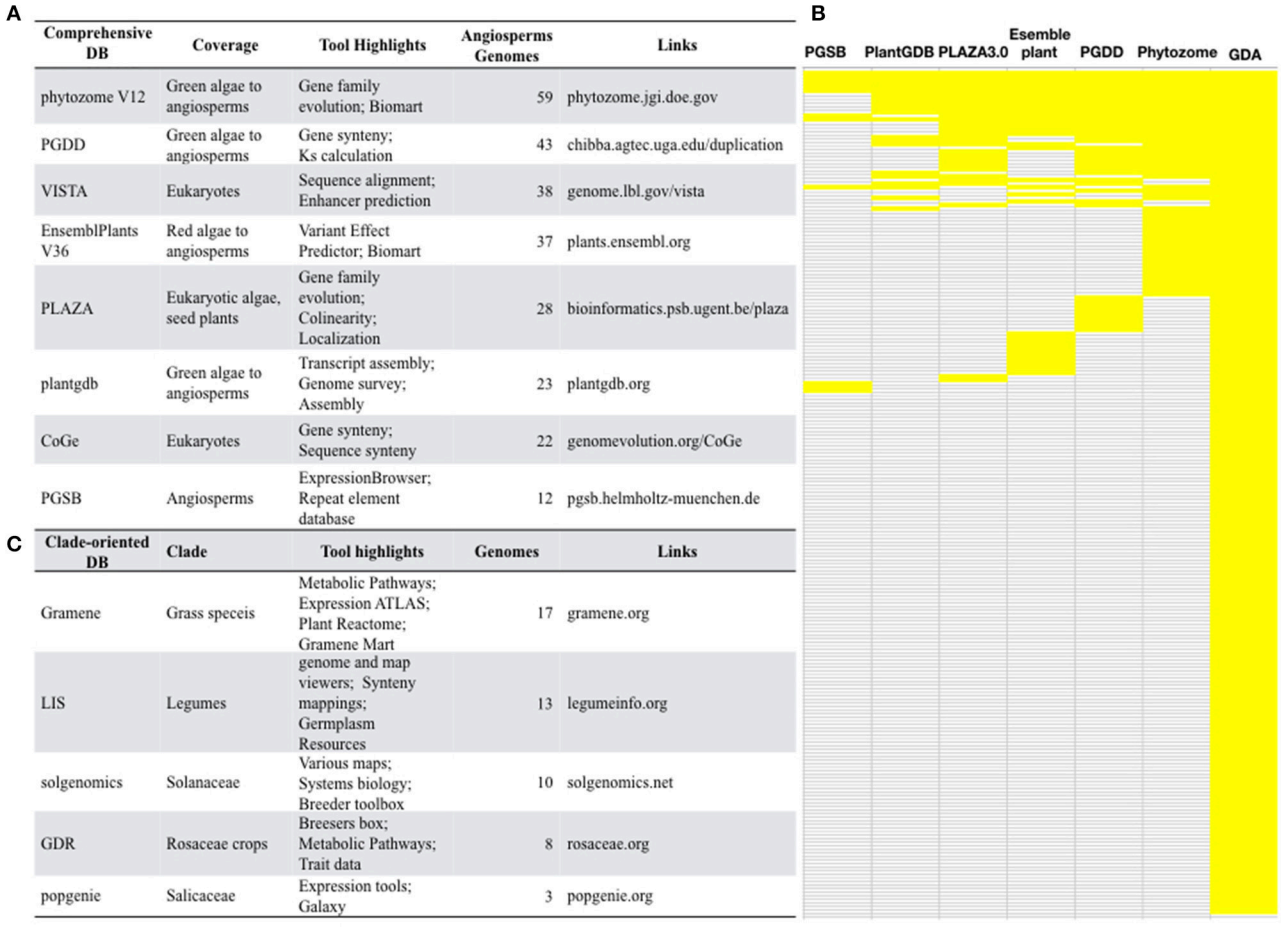
Selected well-constructed genome databases for/covering angiosperms. **(A)** Comprehensive databases and their featured tools and indexed genomes. **(B)** Small-scale genome databases for specific clade of angiosperms. **(C)** A comparison of the comprehensive genome databases. PGSB, Plant Genome and Systems Biology; PlantGDB, Plant Genome Database; PGDD, Plant Genome Duplication Database; GDA, Genome Database for Angiosperms.

With the advances of next generation sequencing (NGS) and the latest third generation sequencing platforms, angiosperms with genome sequences are rapidly accumulating. The Pacific Biosciences (PacBio) company have developed a single-molecule real-time sequencing platform, which outputs long and unbiased reads with average length >10Kb, greatly facilitates the assembly of large and complex angiosperm genomes. The genome sequencing of a desiccation grass *Oropetium thomaeum* (VanBuren et al., 2015), sunflower (Badouin et al., 2017), and quinoa (Jarvis et al., 2017) all relied on PacBio and produced high-quality genome assembly. Comparative analysis of these genomic data allows scientists to answer many important questions of plant biology. Therefore, a high demand for comprehensive genome databases is expected. Currently, several comprehensive databases that include a large collection of plant genomes have been constructed (Figure 2A).

Phytozome (phytozome.jgi.doe.gov) is a large plant genomic portal sponsored by the USA Department of Energy (DOE). The current release of Phytozome (v12) hosts assembled and annotated genomes from 59 angiosperm species, as well as other green lineage species, such as algae, moss, liverworts, selaginella (Goodstein et al., 2012). In addition to BLAST and Gbrowse tools, Phytozome also provide Biomart which allow users to annotate plant gene families, to study the evolution of plant gene families, to display genes in the genomic context (Goodstein et al., 2012), which is valuable for a wide range of scientists who are interested in gene family evolution. However, considering that the genomes of 236 angiosperm species have been sequenced, <1 third of all sequenced angiosperms are included by Phytozome, suggesting the presence of a major gap in the availability of most angiosperm genomes at Phytozome.

The Plant Genome Duplication Database (PGDD) (chibba.agtec.uga.edu/duplication/) is a database currently hosting 43 angiosperm genomes, with tools to identify the intragenome and cross-genome synteny relationships. Synonymous substitutions of homologs inferred from syntenic alignments could be calculated from this database (Lee et al., 2012). By the synteny comparison, PGDD facilitates the identification of evolutionary analysis of gene and genome duplication (Lee et al., 2012).

Ensembl is well-known for developing bioinformatics tools and annotating various eukaryotic genomes (Kersey et al., 2014). The Ensembl Plants (plants.ensembl.org/index.html) provide a HMMER tool for homology searches of gene family members. However, it only covers 37 angiosperm genomes and does not include genome browsers for genomic context views thereby limits its readership.

VISTA (genome.lbl.gov/vista/index.shtml), which includes 38 angiosperm genomes, provides comprehensive tools for analyzing multiple genomes, such as tools for alignment of multiple sequences and large genomic sequences. VISTA has been extensively used by the biomedical community (Poliakov et al., 2014). However, some tools are not applicable to angiosperms as they are restricted to human and mouse data.

Other databases such as PLAZA (Proost et al., 2009), plantgdb (Duvick et al., 2008), CoGe (Lyons, 2008), PGSB (Spannagl et al., 2016) also provide valuable tools for angiosperm comparative genomics. However, none of these databases contains more than 30 plant genomes, which is less than one-eighth of total sequenced angiosperms.

Many of these comprehensive genome databases are well-maintained and are frequently updated with release of new versions, such as PlantGDB V187, Phytozome V12, and Gramene V36. Phytozome updates roughly each year, and PGDD and CoGe update timely upon availability of new genome data. Another feature is that these databases are empowered with various tools other than the open-sourced BLAST and browsers. They offer tools for gene family copies (Phytozome, PLAZA), gene/chromosome synteny (CoGe, PGDD, phytozome, PLAZA, PGSB), protein domains (PlantGDB, Phytozome, EnsemblPlant), gene expression (Phytozome, PGSB), biomart (Phytozome, EnsemblPlants), Intermine (Phytozome), GO annotation (Phytozome, EnsemblPlants, PLAZA, PGSB), alternative splicing (EnsemblPlants, plantgdb).

**Figure 3:**
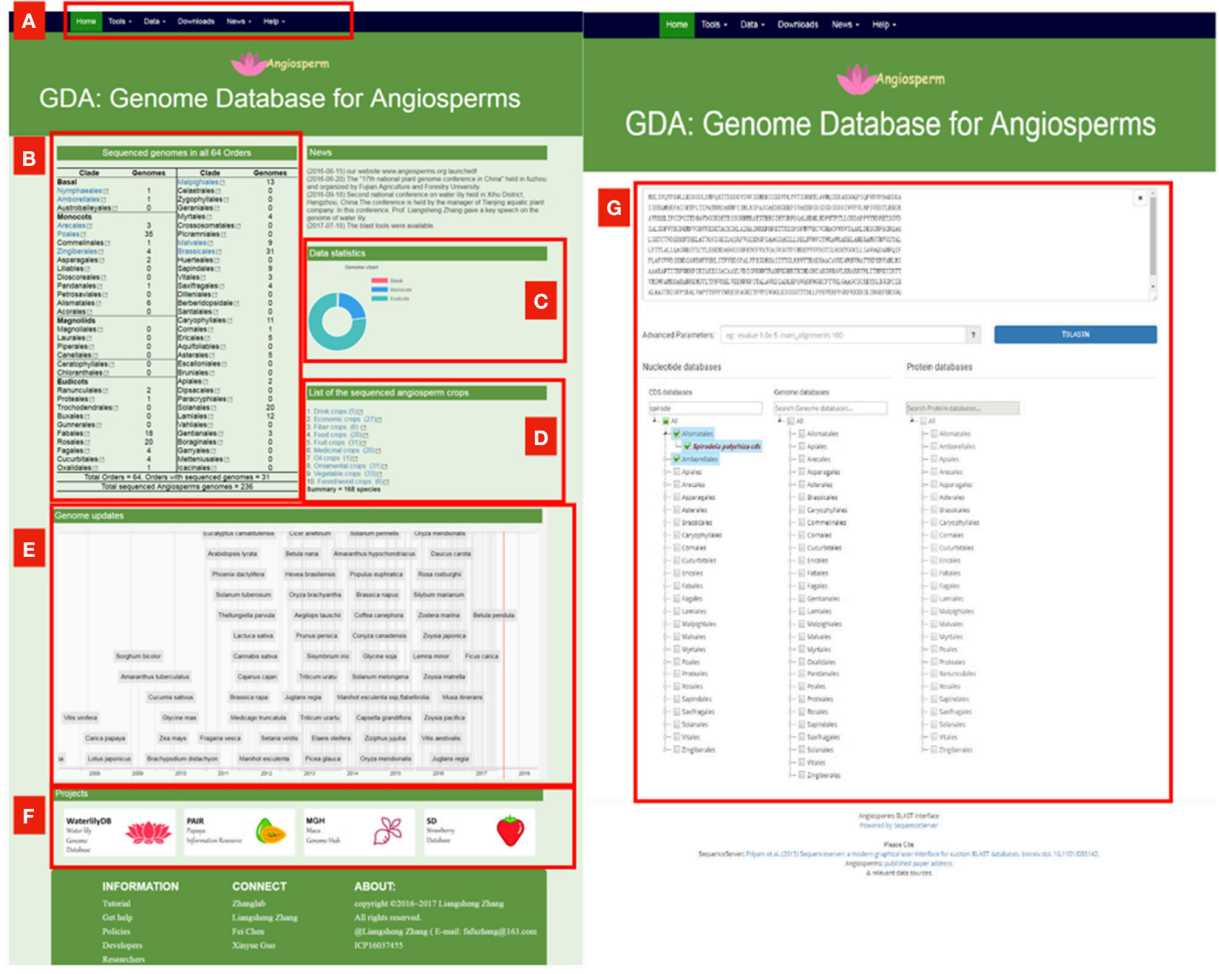
The proposed genome database for angiosperms (GDA, www.angiosperms.org). **(A)** The menu of the database. **(B,C)** Statistics of the sequenced genomes in all the angiosperm orders. **(D)** Statistics of the sequenced crop genomes. **(E)** Timeline for the genome updates and related hyperlinks. **(F)** Ongoing projects for specific angiosperms. **(G)** BLAST page for all the released 235 angiosperm genomes, CDS, and protein information.

Although these comprehensive databases contain a large array of species, the largest one Phytozome only includes 59 angiosperms genomes, accounting for about ¼ of sequenced angiosperm genomes. We constructed the **G**enome **D**atabase for **A**ngiosperms (GDA, www.angiosperms.org) to host all of the released angiosperms genome (Figures 2B, 3). GDA aims to updates all the recently sequenced angiosperm genomes by supplying a timeline (Figure 3A). Currently, all the 236 angiosperm genomes, CDSs, and proteins are provided and can be accessed via BLAST suits and download service.

## Database for clade-oriented angiosperms species

The NGS techniques has significantly accelerated the decoding of genomes. For example, 10 rice species have been sequenced (Table 1). Comparative genomics is a powerful strategy to decode the genetic basis underlying trait evolution and the evolution of genes and genomes. We summarized the five well-constructed clade-oriented genome databases that have a clear goal (Figure 2).

The current version of Gramene (gramene.org) provides curated and integrated genomic information for plants, especially the 17 grass species. Gramene's bioinformatics platforms provide specific softwares for studying the grass traits. Gramene is an early adopter of BioMart (Smedley et al., 2009) and develops the GrameneMart that enables scientists to perform advanced querying, download, and online comparison of grass genomic data sources through a single portal (Tello-Ruiz et al., 2016). Besides the genome framework, Gramene hosts a pathway framework that integrates a plant reactome pathway, and the pathway tool platform “Cyc Pathways,” allowing the fast comparison of grass-specific pathways.

LIS (legumeinfo.org) is the genome information portal for 13 economically important legumes. LIS provides bioinformatics tools for genome and map viewers, and synteny mappings (Dash et al., 2016). LIS also supports the bridge between the genomic information and the crop improvement by supplying the Germplasm Resources. Likewise, Sol Genomics Network (solgenomics.net) is a Solanaceae-oriented database containing genome data, genomic tools, and breeders' tools.

Other clade-oriented genome databases host <10 genomes (Figure 2), such as the Rosaceae crop oriented GDR (Jung et al., 2014), poplar oriented PopGenIE (Sjödin et al., 2009), cool season food legume oriented CSFL (Main et al., 2013). These clade-oriented genome databases gather multiple species, often with economic importance from the same clade, and provide genome data as well as tools for traditional breeders. However, the integration of various genomes needs more frequent updates. The visibility of these databases is often limited to specific scientists, and will be time-consuming for plant kingdom-wide researchers to obtain these data.

These databases are clade-oriented and distinctive in data and tools compared to other databases. They include economically important crops and their related wild species, and contain genome data that are not included in those comprehensive genome database. They usually provide breeding markers such as molecular markers, various maps, breeder's toolbox, primer design, and germplasm resources. Other genomic tools such as BLAST and synteny mapping are useful to visualize and compare the genome data in various scales. Expression visualization often provides large quantity of expression datasets for fast comparison of various genes and gene families. Furthermore, re-sequencing genomes and biological pathway are also provided in these databases. However, the visibility of these databases needs to be improved. They have been frequently accessed by researchers from the same field, but they are less well-known throughout the plant research community. We recommended that a good genome database should be engaged in the alliance, such as Sol and root, tuber and banana (RTB) crops co-sponsored workshop, sharing and co-developing bioinformatics tools.

## Outlook and challenges

### A suit of standards required

First, the transparent operation of genome datasets or tools is required. For data changes in the database, updates need to be recorded, so that we can grasp the new information in a timely manner. This can be published by news and so on. The release of data or tools needs to be forecasted. Solgenomics (solgenomics.net) serves as a good example, as it provides very detailed recent changes to the database. Phytozome also provides genome update in a very conspicuous position.

Second, the genome databases, especially for a single species-oriented ones, require a series of minimum standard tools. Data should integrate the reference but not draft genome, CDS, protein, GFF, GO annotation, and the sequencing quality report. Tools should include the gene search, blast, browse, download.

Third, databases should be maintained for at least 3 years. Good maintenance secures a steady population of users whereas a bad one will only narrow its academic impact.

### Tripal: a toolkit for genome database construction

**Figure 4:**
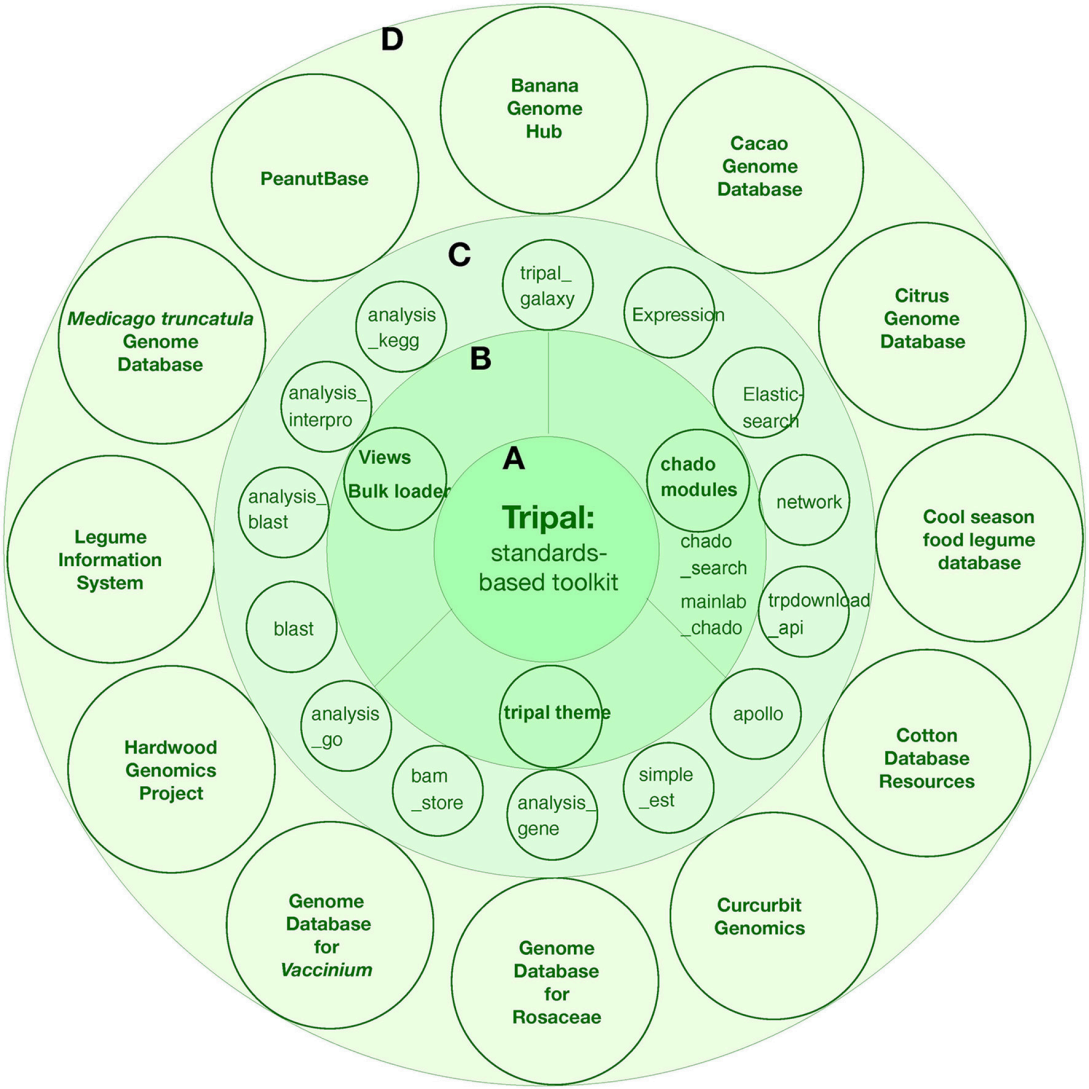
The tripal toolkit for genome database construction. **(A)** The Tripal is a biological application of Drupal. **(B)** core modules of Tripal. **(C)** carious modules developed during the last several years. **(D)** The successful applications of tripal in 12 angiosperm plant projects.

Tripal is a member of the Generic Model Organism Database (GMOD) organization suite of genome tools. The first official version of Tripal was released in 2009 by Stephen Ficklin and Meg Staton at the Clemson University Genomics Institute (CUGI). Tripal incoperates several features: (1) Chado database and related modules for data storage and search; (2) Community-developed modules to help fasten site construction; (3) Provide an out-of-the-box setup for a genomics site for those who simply want to put new genome assemblies and annotations online; (4) Provide Application Programming Interfaces (APIs) for complete customization such that more advanced displays, look-and-feel, and new functionality can be supported; (5) Sites can be customized as desired or using theme packages from drupal. During the last several years, Tripal has been implemented in 12 genome projects, including both single genome centered and multiple genome databases: banana, cacao, citrus, cool season food legume, cotton, curcurbit, rosaceae, Vaccinium, hardwood genomics project, legume information system, Medicago truncatula, and peanut (Figure 4).

### The database facilitates the implementation of the toronto agreement

The papers that reporting genome data usually include (i) genetic and functional changes of the genome and various components such as genes, repetitive elements, and (ii) molecular mechanisms of important traits. However, much information is still not being studied and reported, so the sharing of genomic data raised more concerns. Toronto agreement was proposed in 2009 (Toronto International Data Release Workshop Authors, 2009), aimed to share the scientific data such as the genome sequences before the publication. Data sharing promotes collaboration and contributes to the efficient use of data. Unfortunately, rapid sharing of genome data is still an area that need improvement.

There are a variety of technical means through database to fulfill the Toronto agreement: (i) the establishment of data access thresholds, such as a detailed disclaimer, or the registration system to identify the academic institutions (e-mail address ending with .edu) to share information only to the academic staff; (ii) only provide BLAST and/or Jbrowse and other tools, and DO not provide (it depends and could be optional) download data for data sharing. If only BLAST tools are provided, providers only need to contribute the protein and CDS without the whole genome. Jbrowse could provide genetic information without protein sequence. The provision of data and data sharing on the integrated database is not a nonexistent behavior. The earlier exposed to the public, the earlier intellectual property is committed, and the more efficient it promotes scientific collaborations. At present, due to the requirements by DOE, more genomic data in Phytozome have been released prior to the publication of related genome paper, such as the genome of kalanchoe, monkey flower, and so on. However, the Toronto agreement is yet to be fully implemented and needs in-depth practice.

Because of the value of angiosperms, the large number of genomic data has attracted many scientists and still brings us great challenges: the immediacy, integrity and analytical ability of the data. We provide on GDA database the timeline to update each of the recently sequenced plants (Figure 3). At present, 1,001 *Arabidopsis* strains (Weigel and Mott, 2009), 2,489 millet varieties (db.cngb.org/millet/), and 3,000 rice genomes have completed genome sequencing (The 3,000 rice genomes project). The 10K orchid genome project (J-J Project, sinicaorchid.gzit.net) has been put forward. All these projects made a huge challenge to the current database.

The current database for the processing of such large-scale data also lacks large-capacity computing devices and bioinformatics tools. Visualization of large data also poses a major challenge. The variety of data quality also requires an evaluation system to ensure that low-quality data is filtered to speed up the analysis. In general, the plant genome database will become a new biological branch. The supercomputing equipment, bioinformatics algorithms, and tool development need to be introduced and upgraded. In addition, a user-developer interactive than user-friendly interface is required. Overall, the upgrade of the angiosperm database will greatly enhance our understanding of important issues related to angiosperms and greatly promote the crop breeding process.

## Concluding remarks

The genome data of flowering plants are rapidly accumulating in quantity and complexity. With the big data concept more and more popular in solving big questions, there will be a strong demand to integrate all related data. What's more, bioinformatics tools are usually developed and built firstly in comprehensive or large databases because they attract more researchers. We reviewed and compared the pros and cons on the data, tools, special highlights from three types of genome databases that are mostly used. We also proposed that a comprehensive genome database to host the genomes of all released angiosperms to accelerate the research of major scientific questions at the genome scale.

## Author contributions

LZ: designed the research; FC, WD, and LZ: collected and analyzed the data; FC, WD, JZ, XG, JC, ZW, ZL, HT, and LZ: wrote, revised, and approved the manuscript.

### Conflict of interest statement

The authors declare that the research was conducted in the absence of any commercial or financial relationships that could be construed as a potential conflict of interest.
